# Being Popular or Having Popular Friends, Which Is Better? A Longitudinal Social Network Analysis of Depressive Symptoms among Chinese Adolescents under Major Chronic Stress

**DOI:** 10.3390/ijerph182111164

**Published:** 2021-10-24

**Authors:** Lin Fu, Yue Fan, Jin Cheng, Hao Zheng, Zhengkui Liu

**Affiliations:** 1 Faculty of Humanities and Social Sciences, Beijing University of Technology, Beijing 100124, China; linfu@bjut.edu.cn; 2 CAS Key Laboratory of Mental Health, Institute of Psychology, Chinese Academy of Sciences, Beijing 100101, China; fanyue@psych.ac.cn; 3 Department of Psychology, University of Chinese Academy of Sciences, Beijing 100049, China; 4 Beijing Institute of Education, Beijing 100097, China; chengjin_18@outlook.com; 5 Department of Psychology, University of Alberta, Edmonton, AB T6G 2E9, Canada; hzheng16@ualberta.ca

**Keywords:** friendship networks, sociometric popularity, depressive symptoms, chronic stress, stochastic actor-based model

## Abstract

Background: Previous studies have found that adolescents’ depressive symptoms are influenced by social networks in a stressful context, especially focusing on the processes of social selection and social influence. The current study aimed to explore the coevolution of sociometric popularity and depressive symptoms among adolescents suffering from the stress attached to the Chinese *gaokao*. Methods: The analytical sample comprised 1062 Chinese adolescents who were under significant pressure to return to school for an additional year (returnees) to prepare for college entrance examinations. Students were assessed for depressive symptoms and asked to nominate up to five friends within their classes across four waves (six months). We employed stochastic actor-oriented models to investigate the interdependent relationships between popularity and depressive symptoms. Results: Adolescents’ depressive symptoms negatively predicted future friendship popularity in this stressful situation, but not vice versa. The results of this study also highlighted the importance of friends’ popularity, indicating that adolescents who nominated popular peers as friends tended to subsequently have lower depressive symptoms. Conclusion: These findings suggested that friends’ popularity may serve as a protective factor against depressive symptoms under major chronic stress. Network-based interventions may have practical implications for reducing depressive symptoms under major chronic stress.

## 1. Introduction

Chinese adolescents generally suffer from severe chronic stress due to the annual college entrance exam, which is called the Chinese *gaokao* [1]; this is especially true for senior high school students in their final years [2]. Moreover, some Chinese adolescents suffer from many more stressors than regular high school students under the high stakes attached to the *gaokao*, mainly because they choose to return to cram school for an additional year to prepare for the *gaokao* and to take the examination the following year again since they failed it the first time [3]. These students, known as “senior high school returnees”, tend to place more academic pressure on themselves and have more additional stress attached to the *gaokao*. This decreases the spare time available for relaxing and, hence, is associated with higher risks for mental health disorders than those for their regular peers [4,5].

Adolescence is a particularly vulnerable period for the emergence of depression and other symptoms of distress [6]; hence, Chinese adolescents under the influence of major academic stressors are at higher risk of mental health problems, including depressive symptoms. Depressive symptoms have been confirmed to be associated with a series of negative outcomes for adolescents, such as substance use [7], health-related behaviors [8], and even suicidal ideation [9,10] or suicide attempts [11]. Furthermore, the impacts of major events and adolescent depression may last into adulthood [12,13]. In other words, adolescents are still suffering from the distress of a stressful life and poor mental health even when they finish their academic study and leave for college. Accordingly, the identification of factors that protect against the risk of depression among Chinese adolescents suffering from major chronic stress attached to the *gaokao* is a critical priority for mental health researchers [12,13].

Peer relationships become the main source of social contacts during adolescence [14], and, additionally, bioecological theory suggests that the interplay of adolescents and their peer relationships is particularly relevant for their behavior and development [15]. Multiple empirical studies have demonstrated the fundamental role of peer relationships in determining adolescents’ depressive symptoms [16,17,18,19,20,21,22,23]. Most of these studies were performed within the school context, as school peers provide a primary social environment for adolescents, and they have demonstrated that individuals who are socially integrated have better mental health [18,22]. However, several other studies examined the detrimental effects of being socially integrated on youth mental health [17]. The controversial findings of these studies are partly because of the unique definitions of social status and, more specifically, friendship popularity [24]. For example, some existing research has conceptualized social integration as perceptions of relationship quality or perceived social status [25,26], whereas others have described the structural aspects of social integration from a social network perspective [22,27]. Since the social network perspective focuses on a system of peer relationships and provides conceptual and analytical tools for describing individuals’ positions in a friendship network, it not only captures the information on direct and proximal but also indirect and distal friendships within a specified network (e.g., class or clique). Taken together, we adopt a social network perspective to examine the effects of social status, measured by friendship network popularity, on depressive symptoms among Chinese adolescents who are suffering from major chronic stress attached to the *gaokao*.

Friendship network popularity is an emergent property characterizing one’s social status in the school context, indicating the extent to which an adolescent is nominated by friends or is liked by his or her peers. There is increasing evidence indicating the protective effects of friendship network popularity in determining adolescents’ health [28,29], substance use [30], and internalized problems such as depressive symptoms [18,27,31]. These studies using structural definitions of friendship network popularity suggested that higher friendship network popularity was associated with fewer depressive symptoms in adolescence [27,31,32]. In addition, it is necessary to examine gender differences because research has demonstrated that gender may play a role in friendship network popularity and in depressive symptoms [27,32].

However, there is still a relative shortage of studies investigating the interdependent relationships between friendship network popularity and adolescents’ depressive symptoms. Adolescent relationships are dynamic, as adolescents tend to lose and gain friends every school year [33,34], and the popularity of friends in their network also changes dynamically even within a short time. In recent decades, an increasing number of studies have described network and behavior dynamics (e.g., depressive symptoms) using stochastic actor-based models [35,36,37,38]. This simulation-based approach capitalizes on longitudinal sociocentric network data, and a benefit of this approach is that it explicitly models the interconnectedness and temporality inherent to social life rather than making post hoc adjustments for the statistical dependence of observations (a limitation of regression-based approaches) [39,40]. Accordingly, the stochastic actor-based model could provide an opportunity to explore the mechanism between peer popularity and adolescents’ depressive symptoms in a longitudinal design in the current study.

Although the fact that indirect relationships can also affect adolescents’ mental health has already been confirmed theoretically [41] and empirically [42], researchers have barely recognized that one’s friends’ popularity has the ability to influence one’s depressive symptoms. Only Peters and colleagues (2010) found that the popularity of an adolescent’s best friends was associated with one’s health behaviors [43]. Given the scarce evidence of the association between friends’ popularity and their depressive symptoms, circumstantial evidence should also be taken into account. First, from a social network perspective, having a popular friend would potentially enhance one’s network centrality, which has been found to be negatively associated with depressive symptoms [25]. Second, a higher level of popularity, which is found to be associated with a decrease in depressive symptoms, may also affect their connected friends. We already know that similar levels of depressive symptoms tend to be found between connected friends [41] due to a social influence mechanism [35]. It is reasonable to believe that forming ties with popular friends who may have lower levels of depressive symptoms would also protect oneself from the risk of depressive symptoms.

Therefore, this study aimed to examine and explore the relationships between one’s popularity and depressive symptoms and between one’s friends’ popularity and depressive symptoms among Chinese late adolescents in the context of high stress exposure. By applying stochastic actor-based models based on longitudinal network data, three research questions were addressed in the current study. First, we examined the interdependent relationships between friendship network popularity and adolescents’ depressive symptoms in the stressful context of the *gaokao*. Consistent with previous studies [38], it was anticipated that among adolescents suffering from major stress attached to the *gaokao*, high levels of depressive symptoms would predict decreasing levels of popularity over time, but not vice versa. Second, we explored whether the popularity of an adolescent’s peers has an impact on the level of his or her depressive symptoms. Given the circumstantial evidence mentioned above, we expect that having connections with popular friends may help to decrease the risk of depressive symptoms. Third, we also investigated two processes of social selection and the social influence of depressive symptoms that still exist in a stressful context among Chinese adolescents. Due to the lack of relevant studies providing evidence of the chronic stress affecting adolescents in a relatively short period of time, we refrained from predicting any significant social selection or social influence effects of depressive symptoms in this study.

## 2. Materials and Methods

### 2.1. Participants

At Jin’an cram school, which is located in the town of Maotanchang in Anhui Province, commonly known as the largest test-prep factory in China, almost fifteen thousand senior high school returnees, or three times the town’s official population, prepare for the *gaokao* in their year of cramming every year. These returnees come from all provinces of China, and the reason why they and their parents choose Maotanchang is mainly because it is famous for its success in raising students’ *gaokao* scores and its military-style of management that helps to ensure success. Every year, approximately two-thirds of returnees who study in Maotanchang town are accepted into universities (almost 95% of them did not receive high enough scores in the *gaokao* the previous year). Students there have to arrive at their classroom at 6:20 am and end their last class at 10:50 pm, attending more than 12 classes per day. All types of electronic products, including cellphones and laptops, are forbidden in the school, and all forms of entertainment in this town, such as internet cafes, have been shut down by the local government. Accordingly, the stressful, anxious, and depressing atmosphere for students in Maotanchang, especially for returnees, is due to multiple factors. Students in the town can do nothing but study there. Despite the school environmental factors, there are still stressors from the students themselves and their families. Most students come from rural areas, and they pin all their hope on the *gaokao* to be enrolled in a quality university and live a better life. They even carry the hope of changing their whole family’s fortune. Failing the test once makes this more stressful with no doubt, as preparing for the next test requires not only an amount of money, but a whole year’s worth of time and effort day after day. Currently, this typical phenomenon of Maotanchang in China has attracted extensive attention from the international community. Larmer (2014) wrote an article with a comprehensive and detailed description about Maotanchang and Jin’an cram school, which was published in New York Times [44].

The participants in the present study were 1062 Chinese adolescents from seven classrooms in the Jin’an cram school who participated as the control group in a network-based mental health intervention program. In the intervention group, the relationships between friendship networks and depressive symptoms and other variables of interest may have been affected as a consequence of a network-based intervention; thus, only the control group classes were included in the current study. The numbers of valid data points for the four waves were 1006, 1013, 959, and 866. After the inclusion of adolescents nominated in these classes who did not offer valid data in each of the four waves, the final network sample consisted of 1062 late adolescents (528 females, 532 males, 2 missing values), with an average age of 18.50 years (SD = 0.83) and a range from 16 to 22 years as of September 2018. Complete descriptions of the demographic information of the participants can be found in Table 1. Attrition analyses showed no significant differences in the average number of nominated friends, the average scores for depressive symptoms, and perceived social support in the first wave between students who participated in all four assessments (755, 71.1%) and those who participated in fewer than four assessments (307, 28.9%).

### 2.2. Procedure

The study was approved by the ethics review committee at the authors’ institute. Data were collected four times: September 2018 (T1; two weeks after the beginning of the first semester of the academic year), October 2018 (T2), December 2018 (T3), and March 2019 (T4; four weeks after the beginning of the second semester). The time intervals between each measurement point ranged from 1.5 to 3 months.

Based on convenience, students in ten classes were chosen to complete the questionnaires across the four waves. Of the ten classes, two were eliminated for subsequent selection as the intervention group, and another class was eliminated because of their high rates of missing data (>50%). Therefore, the remaining seven classes constituted the subjects in the current study. Oral agreement from the head teachers from the chosen classes was obtained after they were informed about the aim of this survey and the amount of time required to complete it (an average of 20 min each time). Seven head teachers were trained as instructors in the data collection processes by a doctoral candidate using the same standardized instructions. The data collection took place in each classroom on the same day during school hours and was administered by the head teacher of each class. Respondents were guaranteed that their responses would be kept confidential and be used only for scientific research purposes and that they had the right to withdraw from the research at any time without consequences. After ensuring that the students understood this completely, they signed written informed consent forms before volunteering to answer the questions. The students could ask any questions while filling out the questionnaires.

### 2.3. Measures

*Sociodemographic variables.* Demographic information was obtained at T1 using a self-report questionnaire with multiple response options for the questions, including gender, age, whether the student was an only child (1 = *yes*, 2 = *no*), family address (1 = *urban*, 2 = *rural*), maternal educational level (1 = *never been to school*, 2 = *primary school*, 3 = *junior high school*, 4 = *senior high school*, 5 = *college or above*), and subjective socioeconomic status (“How would you describe your family’s economic status in the area?”, 1 = *low*, 2 = *below average*, 3 = *average*, 4 = *above average*, 5 = *high*).

*Peer networks.* Network ties were measured by sociometric data in the form of friendship nominations collected from Wave 1 to Wave 4. Specifically, each returnee was asked to nominate up to five friends in his or her class by writing each friend’s full name on the questionnaire and providing information on gender that was then matched to student rosters. The entire network of a class could be constructed only when the sender (nominator) and receiver (nominee) attended the same class.

*Depressive symptoms.* Depressive symptoms were assessed using the brief form of the Center for Epidemiological Studies Depression Scale (CES-D). The Brief CES-D is a 10-item self-report measure of depressive symptoms. Participants identified how often they had experienced depressive symptoms (e.g., “I was bothered by things that usually don’t bother me”) over the past week by providing a rating on a 4-point Likert-type scale from 0 (rarely: less than once per day) to 3 (most or all the time; 5–7 days). Total scores ranged from 0 to 60, with higher scores indicating higher levels of depressive symptoms. The Chinese version of this scale has been validated and utilized extensively with Chinese adolescents [45]. In the current study, the CES-D was also found to have good internal consistency (Cronbach’s α ranged from 0.79 to 0.83 for all waves) for late adolescents experiencing major life events. Given the requirements of SIENA that dependent behavioral variables must have nonnegative integer values and that the number of different values should be less than ten [46], the scores for depressive symptoms were z-standardized within the sample of seven classes and subsequently transformed into a 7-point (0–6) ordinal scale using increments of one standard deviation of the continuous Z-score as cutoff points.

### 2.4. Statistical Analyses

Analyses were conducted with stochastic actor-based models [39,40,46] that are capable of handling the interdependent nature of network data by simultaneously estimating network changes (e.g., whether an adolescent creates, sustains, or dissolves a friendship with others) and behavior changes (e.g., whether an adolescent becomes more or less depressed) in a complete network (e.g., all adolescents within one class). Through an iterative simulation procedure within the Markov chain Monte Carlo approach [39,40], the model estimates were obtained to explore the bidirectional relationships between the network and depression over time. Missing data due to participant nonresponse were addressed by coding outgoing friendship ties as missing for adolescents who still attended the class but did not complete a survey. In addition, those who joined and left the class during any period were coded as joiners or leavers of friendship networks [46]. All seven classes were combined in one model but analyzed separately by applying the multigroup option to gain sufficient power, since we aimed to explore the general pattern rather than by examining the variance between classrooms. The current study used RSiena version 1.2 [46], which is a package contributed by SIENA for the statistical system R [47].

In the current study, theoretical considerations and established backward model selection procedures were used to determine whether the predictors significantly contributed to the overall model according to the guidelines for the stochastic actor-based model introduced in previous studies [39,40]. Three sets of effects were included to estimate the longitudinal coevolution of the peer network and depressive symptoms. First, various structural effects of the friendship networks were described in the preliminary analyses of the model to show the basic tendencies of network development in the context of high stress (e.g., *outdegree*, *reciprocity*, *transitive triplets*, *3 cycles*). Additionally, two pairs of degree-based effects were added to the original network structural model, including one pair of *degree-related popularity* and another pair of *degree-related activity* (indegree and outdegree). *Indegree-related popularity* and *outdegree-related popularity* were retained in the final model because of their significance. A full list of all parameters included in the final model and their interpretations are provided in Table 2.

The second set of parameters explored the network dynamics by estimating the effects of depressive symptoms on network changes. Three depression-related parameters were added to the model: the depression ego effect, the depression alter effect, and the depression similarity effect. The *depression ego effect* captures the effect of depressive symptoms on the number of outgoing ties an adolescent nominates while controlling for the number of friends who nominated them. However, the *depression alter effect* captures the effect of depressive symptoms on the number of incoming friend nominations. Moreover, the *depression similarity effect* refers to social selection based on similarity in levels of depressive symptoms. The *depression ego effect* is deleted in the final model, while the *depression alter effect* is retained, despite its insignificance, because it is the focal parameter of network dynamics (social selection) in the current study. Additionally, three *gender-related effects* were added to the original model in a backward selection procedure, during which the significant effects were retained as control variables for further analyses.

Finally, the third set of parameters examined the behavior dynamics by estimating the effect of peer network characteristics on individuals’ depressive symptoms. Three effects, namely, the *average similarity effect*, *popularity ego effect,* and *popularity alter effect,* were retained in this part of the final model during the backward procedure. The *average similarity effect* referred to the tendency of adolescents to adopt the level of depressive symptoms of friends with whom they were connected. This effect was not eliminated from the model during backward selection because it is the core social influence effect that we focused on. The *popularity ego effect* was the effect of an individual’s popularity on his or her depressive symptoms compared with others. The *popularity alter effect* revealed how the average popularity (indegree) of friends to whom an adolescent is tied affects his or her depressive symptoms. Gender was included as a control variable in this part.

## 3. Results

### 3.1. Descriptive Statistics

Table 3 provides the descriptive results regarding peer network properties and depressive symptoms in each wave and their changes in each period. The average numbers of friend nominations of the seven classes were 250, 281, 266, and 236 from T1 to T4, indicating a tendency to increase initially and then decrease. The mean density was 0.02 for every wave. The descriptive results for the reciprocity index suggest that more than 60% of the friendship nominations were reciprocated at T1 and that the reciprocity index declined to 40% at T4, indicating a tendency to decrease over time. Moreover, the mean scores of depressive symptoms for the four waves indicated an increasing trend as the *gaokao* approached, which is also supported by the *linear shape effect* in the behavioral dynamics of SIENA models. The significant Moran’s *I* coefficients (network autocorrelation coefficients) were 0.04–0.08, suggesting that adolescents tend to have levels of depressive symptoms similar to those of their friends [48]. Additionally, the Jaccard indexes between consecutive waves were 0.33–0.28 with a tendency to decrease, which demonstrates that the stability of the peer network is relatively low but acceptable [40].

### 3.2. SIENA Model

#### 3.2.1. Structural Effects

The results related to network structures are provided in Table 4. *Outdegree*, *reciprocity*, *transitive triplets*, and *3 cycles* are four parameters that represent endogenous network dynamics. Adolescents had a decreasing number of friendships formed with anyone in the class, as indicated by the significantly negative *outdegree* parameter (*β* = −3.69, *OR* = 0.02, *p* < 0.001). The positive *reciprocity* parameter suggests that individuals prefer to reciprocate friendship nominations (*β* = 2.58, *OR* = 13.25, *p* < 0.001), and the significantly positive *transitive triplets* effect indicates that they tend to become friends with adolescents to whom their friends are already connected (*β* = 0.76, *OR* = 2.13, *p* < 0.001). The *3 cycle* parameter is negative and statistically significant (*β* = −0.61, *OR* = 0.54, *p* < 0.001), indicating that an individual does not tend to form ties with the person who nominated his or her nominator. Two additional structural features of peer networks described in Table 4 are the *indegree-related popularity effect* (*β* = 0.26, *OR* = 1.30, *p* < 0.001), which indicates that peers receiving many friendship nominations tend to become more popular, and the *outdegree-related popularity effect* (*β* = −0.54, *OR* = 0.58, *p* < 0.001), which suggests that individuals nominating more friends have a lower probability of receiving subsequent friendship nominations.

#### 3.2.2. Depressive Symptom Effects on Friendship Networks

The results associated with friendship selection and depressive symptoms are provided in Table 4. As shown, only one of three possible focal parameters related to depressive symptoms is significant. The significant alternating *depression effect* (*β* = −0.03, *OR* = 0.97, *p* = 0.03) suggests that one’s depressive symptoms are negatively associated with the number of nominations received by an individual, indicating that one’s popularity may be affected by his or her depressive symptoms. In other words, more depressed adolescents had a higher likelihood of becoming unpopular than their less depressed peers. At the same time, the *depression similarity effect* emerged as nonsignificant (*β* = 0.13, *OR* = 1.14, *p* = 0.15), which suggested that there is no tendency for adolescents to select friends who have similar depressive levels. Additionally, three significant *gender-related effects* are retained in the final model. The negative and significant *gender ego effect* suggests that male adolescents tend to nominate more friends, whereas the positive *gender alter effect* indicates that female adolescents tend to be nominated as friends more often. The positive *gender similarity effect* reveals a preference for adolescents to form friendships with those of the same gender.

#### 3.2.3. Friendship Network Effects on Depressive Symptoms

The results of the influence effects of the model are described in Table 4. First, the significantly positive *linear shape of depression symptoms* demonstrates an overall average tendency toward high levels of depression (*β* = 0.58, *OR* = 1.78, *p* < 0.001). The negative *quadratic shape effect* means that over time, adolescents with higher depressive scores are more likely to experience decreased depressive symptoms, whereas students with lower depressive scores are likely to report increasing depressive symptoms (*β* = −0.32, *OR* = 0.72, *p* < 0.001). Furthermore, two of the three focal parameters are not statistically significant. The *average similarity effect* indicates that the process of social influence is not significant (*β* = −0.49, *OR* = 0.61, *p* = 0.24); thus, there is no influence effect between individuals in the context of major stress. The result regarding the *popularity ego effect* is also nonsignificant (*β* = 0.00, *OR* = 1.00, *p* = 0.46), suggesting that one’s popularity does not have an impact on his or her own depressive symptoms. The negative and significant *popularity alter effect* indicates that adolescents may become less depressed if they have connections (both giving and receiving nominations) with those with a high indegree (popular) in the network (*β* = −0.08, *OR* = 0.92, *p* = 0.03). Finally, females reported lower levels of depressive symptoms (*β* = 0.10, *OR* = 1.10, *p* < 0.05).

## 4. Discussion

This longitudinal study examined the bidirectional relationships between friendship popularity and depressive symptoms among Chinese adolescents in the context of high stress related to the *gaokao* across four waves using stochastic actor-based models. The results indicated that depressive symptoms had an impact on adolescents’ popularity, but not vice versa. Another novel finding was that nominating a sociometric popular friend instead of being popular oneself within peer networks would help adolescents lower their level of depressive symptoms. However, neither a social selection process based on depressive symptoms nor a social influence process between connected individuals’ depressive symptoms was found in the current sample.

The network and behavioral dynamics of the model showed that depression symptoms predicted the future popularity of adolescents within high-stress peer networks, whereas one’s level of popularity in the peer networks had no significant relationships with his or her subsequent depressive symptoms. Specifically, more depressed adolescents tended to receive fewer nominations, while less depressed individuals tended to subsequently become more popular. The evidence of negative associations of adolescents’ popularity status and their depressive symptoms has been provided in a cross-sectional study [25]. The results of the current study help to further clarify this pattern by demonstrating that higher levels of depressive symptoms are the main contributors rather than causes of adolescents’ lower popularity status in the stressful context related to the *gaokao*. In the large and sparse network in the current study, depressed adolescents tended to become marginalized within their network because of their withdrawal from social connections, which is consistent with the results of previous studies in other contexts [37,38]. Moreover, less depressed adolescents may receive more friendship nominations because they are relatively active in their social life within peer networks. Furthermore, inconsistent with previous studies [32], an effect of popularity on depressive symptoms was not found in the current research. This is probably because popularity based on personal likability used in the current study is different from the perceived popularity based on social status used in most existing mental health studies [24,49].

The novel finding of the behavior dynamics of the statistical model is that under stressful circumstances related to the Chinese *gaokao*, the average popularity of friends with whom an adolescent is connected will affect his or her depressive symptoms, whereas one’s popularity status will not. Specifically, forming ties to popular peers (peers with a high indegree) helps to decrease one’s future depressive symptoms. Individuals who have ties with more popular adolescents usually appear to have more friends of friends (friends with two degrees of separation), thereby enhancing their centrality positions within the entire network to some extent. Given that some previous studies revealed that people on the periphery of a network usually have significantly worse CES-D scores and have fewer friends [25], individuals who have more ties to popular individuals within the network may have lower depressive symptoms because of reinforcing support from the network system (not only direct ties). An alternative explanation is that popular adolescents may have certain personality traits that contribute to the mental health of their friends [50,51]. Accordingly, having more popular friends could play an active role in reducing the depressive symptoms of adolescents suffering from major chronic stress related to the *gaokao*.

Although there are cross-sectional associations between peer network relationships and adolescents’ depressive symptoms, as indicated by the results of *Moran’s I*, the stringent causal methods provided neither evidence of a significant social selection process nor a social influence process over time from several large classroom-based peer networks in this sample. This result is not in accordance with those of several previous studies that found social influence or social selection mechanisms for depressive symptoms within smaller peer networks in a regular high school context [35,38]. First, the possible interpretation regarding why the social selection process was not found in the current sample is that adolescents under the high stakes attached to the *gaokao* tend to have little time and few opportunities to choose friends according to their preference. Since they are strangers to each other, they probably start a friendship by chance. Given that only the formation stage of peer networks was observed in the current study, adolescents did not have sufficient time to build and maintain long-term friendships. Therefore, it is difficult for researchers to identify a tendency toward social selection based on depressive symptoms in a relatively short time span. This is consistent with the findings of some previous studies that also collected their data in a short time span, such as three months [36]. Second, the absence of a social influence process in this study is possibly due to the ceiling effect of adolescents’ depressive symptoms caused by academic stress. Social influence effects may disappear when the individuals in their networks have relatively high levels of depressive symptoms. Most existing studies were conducted in regular middle or high school settings [35,37,38], while our study was among the first to test this assertion in networks of adolescent peers suffering from major chronic stress attached to the *gaokao*.

Our analytical technique makes it possible to describe the development of peer networks. There is a significant tendency among adolescents to send fewer ties over time but to reciprocate friendship ties and form transitive structures that may be more supportive of them under pressure. This is in accordance with many previous SIENA studies estimating adolescents’ school-based peer networks [52,53,54,55]. The decline in the absolute number of reciprocity relationships in friendship networks in the current study may result from the dissolution of ties with weak reciprocity, individuals leaving the networks, and the social reluctance induced by academic stress. Moreover, the significant indegree-related popularity effect suggested that receiving many friendship nominations is a self-reinforcing process in the current context, which may result from the fact that adolescents tend to nominate those who are as popular as them or more popular than them [56]. Finally, consistent with recent SIENA studies [35,36,38,57], this study revealed that adolescents tend to form friendships with same-sex peers.

This study provides further evidence for a dynamic relationship between friendship network popularity and depressive symptoms in a stressful context by emphasizing the importance of friends’ popularity. First, to our knowledge, this is the first study to examine the interrelations between peer popularity and depressive symptoms using social network analysis. The current findings help to further understand the interrelationships of social status and mental health. Next, returnees in Maotanchang high school are representative groups who barely have any social life outside classes and have to deal with increased amounts of academic stress, which provided researchers with a typical sample of peer networks under major chronic stress. Moreover, this study identified friends’ popularity as a protective factor against adolescents’ depressive symptoms under major chronic stress. The fact that friends’ average popularity tends to have a protective effect on adolescents’ depressive symptoms may be of particular interest for teachers, social workers, counselors, and psychologists who are working with adolescent populations. Providing opportunities for adolescents to form more new ties with popular peers may help them enhance their friends’ average popularity and decrease their risks of being depressed under situations of major chronic stress. Accordingly, adopting network-based interventions would be more appropriate and efficient than providing psychological services to adolescents individually when planning efforts to prevent depression under academic stress from high school.

This study had several limitations that should be considered. The peer network and depressive symptoms of adolescents were assessed at four time points within a six-month period, which does not capture much of the change that occurs during a full school year, especially in the last few months, as the last time point of data collection was three months before the *gaokao*. The main reason why we did not collect more data in more frequent assessments or subsequent time points is that returnee students from Maotanchang high school are constantly focused on achieving good *gaokao* scores and, hence, have little time to do anything but study, especially as the exam approaches. Moreover, it would have been more informative to consider additional variables. Variations in individuals’ classrooms and their own families, such as the inclusiveness of head teachers and the supportiveness of family members, could play a role in adolescents’ attitudes and behaviors in social interaction, thereby conditioning the formation and development of peer networks under situations of academic stress. Another limitation of our research is the representativeness of the samples. Our main purpose was to explore the potential relationships of network dynamics and depressive symptomology in a context with major chronic stress. Our choice of a typical sample suffering from major chronic stress was late adolescents in China from a cram school studying for the *gaokao*. Thus, our results cannot be generalized beyond students’ classroom-based friendships. Future research that incorporates alternative samples in stressful contexts would add to the literature on peer networks and mental health in the context of major chronic stress.

## 5. Conclusions

In conclusion, by applying social network methodology, the current study demonstrated that adolescents’ popularity is more a consequence of rather than a contributor to depressive symptoms. This study also highlighted the importance of friends’ popularity in reducing the risk of depression for adolescents under stress. More generally, these findings of the present study emphasize that network-based interventions should be considered an effective way to prevent adolescents’ depression in a stressful context.

## Figures and Tables

**Table 1:** Participants’ demographic information.

Variables		N (%)
Gender		
	male	496 (49.3)
	female	510 (50.7)
Age		
	M (SD)	18.46 (0.83)
Only child		
	yes	327 (32.5)
	no	647 (64.3)
	missing value	32 (3.2)
Residence		
	urban	347 (34.5)
	rural	629 (62.5)
	missing value	30 (3.0)
Maternal education level		
	never	64 (6.4)
	primary school	367 (36.5)
	junior high school	354 (35.2)
	senior high school	122 (12.1)
	college or above	69 (6.9)
	missing value	30 (3.0)
Socioeconomic status		
	low	38 (3.8)
	below average	188 (18.7)
	average	671 (66.7)
	above average	74 (7.4)
	high	3 (0.3)
	missing value	32 (3.2)

Note: Data from the first wave.

**Table 2:** Interpretation of the parameters included in the stochastic actor-based model of the peer networks and depressive symptoms of adolescents under chronic stress.

Parameter		Interpretation
**Network Dynamics**		
	Outdegree	Tendency of actors to have outgoing ties
	Reciprocity	Tendency of actors to reciprocate a friendship tie
	Transitive triplets	Tendency of actors to form transitive triadic patterns of relationships
	3 cycles	Tendency of actors to form cyclic triadic patterns of relationships
	Indegree-related popularity	Effect of having indegree nominations on subsequent number of ingoing ties
	Outdegree-related popularity	Effect of having outgoing ties on subsequent number of ingoing ties
	Gender alter effect	Effect of gender on number of ingoing ties
	Gender ego effect	Effect of gender on number of outgoing ties
	Gender similarity	Tendency to become friends with individuals of the same gender
	Depression alter effect	Effect of depressive symptoms on the number of ingoing ties
	Depression ego effect	Effect of depressive symptoms on the number of outgoing ties
	Depression similarity	Tendency to become friends with individuals of similar levels of depressive symptoms
**Behavior Dynamics**		
	Depression linear shape	Overall linear tendency of depressive symptoms
	Depression quadratic shape	Overall quadratic tendency of depressive symptoms
	Average similarity effect	Tendency of actors to adopt the level of depressive symptoms of friends with whom they were connected
	Popularity ego effect	Effect of individuals’ popularity on depressive symptoms
	Popularity alter effect	Effect of average indegrees of nominated friends on one’s depressive symptoms
	Effect of gender	Effect of gender on depressive symptoms

**Table 3:** Descriptive results for depressive symptoms and peer network changes.

		Wave 1		Wave 2		Wave 3		Wave 4
**Social networks**								
	Average ties	250		281		266		236
	Average outdegree	2.40		2.54		2.31		1.96
	Density	0.02		0.02		0.02		0.02
	Reciprocity Index	0.63		0.54		0.48		0.41
**Depressive symptoms**								
	Mean (SD)	19.14 (4.97)		21.17 (5.31)		21.95 (5.34)		22.59 (4.84)
	Moran’s I (p)	0.04 (0.030)		0.08 (0.000)		0.05 (0.003)		0.07 (0.001)
			**Wave 1–2**		**Wave 2–3**		**Wave 3–4**	
**Social network changes**								
	Average dissolved ties		186.29		171.29		142.86	
	Average emerged ties		199.71		179.43		155.00	
	Average maintained ties		178.43		214.71		207.86	
	Leavers		6		9		55	
	Joiners		13		2		6	
	Jaccard index (stability)		0.33		0.30		0.28	

**Table 4:** The stochastic actor-based model of the peer networks and depressive symptoms of adolescents under chronic stress.

		*Β*	*SE*	*t*	*OR*	*p*-Value
**Network Dynamics**						
	Outdegree	−3.69	0.08	−44.44	0.02	<0.001
	Reciprocity	2.58	0.05	56.92	13.25	<0.001
	Transitive triplets	0.76	0.02	30.96	2.13	<0.001
	3-cycles	−0.61	0.05	−11.86	0.54	<0.001
	Indegree-related popularity	0.26	0.03	7.66	1.30	<0.001
	Outdegree-related popularity	−0.54	0.04	−13.99	0.58	<0.001
	Gender alter effect	0.20	0.05	4.21	1.22	<0.001
	Gender ego effect	−0.18	0.05	−3.49	0.84	<0.001
	Gender similarity	1.37	0.05	28.05	3.92	<0.001
	Depression alter effect	−0.03	0.02	−1.86	0.97	0.031
	Depression similarity (social selection)	0.13	0.13	1.04	1.14	0.148
**Behavior Dynamics**						
	Depression linear shape	0.58	0.13	4.56	1.78	<0.001
	Depression quadratic shape	−0.32	0.04	−8.94	0.72	<0.001
	Average similarity (social influence)	−0.49	0.68	−0.72	0.61	0.235
	Popularity ego effect	0.00	0.02	−0.11	1.00	0.456
	Popularity alter effect	−0.08	0.04	−1.93	0.92	0.027
	Effect from gender	0.10	0.04	2.22	1.10	0.013

## Data Availability

All data included in this study are available upon request by contact with the corresponding author.

## References

[1] Yu S., Chen B., Levesque-Bristol C., Vansteenkiste M. (2018). Chinese Education Examined via the Lens of Self-Determination. Educ. Psychol. Rev..

[2] Roberts D., Zhao J. (2016). This Is How China Preps for the Big Test. Bloom. Bus..

[3] Wu Q., Cui S. (2019). Is the Restudy Experience for the National College Entrance Examination a Barrier to Student’s Career Development? —Evidence from Beijing College Students Panel Survey. Educ. Econ..

[4] Feng Y.C., Ding L. (2007). Investigation of the Mental Health Status of 174 Returnees from Senior High School Graduate before the College Entrance Examination. Chin. J. Health Psychol..

[5] Wu H., Xiao R. (2010). Study of Life Quality among Returnees from Senior High School Graduates. Chin. J. Health Psychol..

[6] Andersen S.L., Teicher M.H. (2008). Stress, sensitive periods and maturational events in adolescent depression. Trends. Neurosci..

[7] Lo C.C., Cheng T.C., de la Rosa I.A. (2015). Depression and substance use: A temporal-ordered model. Subst. Use Misuse.

[8] Testa C.R., Steinberg L. (2011). Depressive symptoms and health-related risk-taking in adolescence. Suicide Life-Threat. Behav..

[9] Batterham P.J., van Spijker B.A.J., Mackinnon A.J., Calear A.L., Wong Q., Christensen H. (2018). Consistency of trajectories of suicidal ideation and depression symptoms: Evidence from a randomized controlled trial. Depress. Anxiety.

[10] Ellis T.E., Trumpower D. (2008). Health-risk behaviors and suicidal ideation: A preliminary study of cognitive and developmental factors. Suicide Life-Threat. Behav..

[11] Nanayakkara M.D., Misch M.D., Chang D.O., Henry D. (2013). Depression and exposure to suicide predict suicide attempt. Depress. Anxiety.

[12] Hertzman C., Boyce T. (2010). How experience gets under the skin to create gradients in developmental health. Annu. Rev. Publ. Health.

[13] Johnson D., Dupuis G., Piche J., Clayborne Z., Colman I. (2018). Adult mental health outcomes of adolescent depression: A systematic review. Depress. Anxiety.

[14] Bokhorst C.L., Sumter S.R., Westenberg P.M. (2010). Social Support from Parents, Friends, Classmates, and Teachers in Children and Adolescents Aged 9 to 18 Years: Who Is Perceived as Most Supportive?. Soc. Dev..

[15] Bronfenbrenner U., Morris P.A., Damon W., Lerner R.M. (2006). The ecology of developmental processes. Handbook of Child Psychology Volume 1: Theoretical Models of Human Development (Fifth).

[16] Bearman P.S., Moody J. (2004). Suicide and friendships among American adolescents. Am. J. Public Health.

[17] Falci C., McNeely C. (2009). Too many friends: Social integration, network cohesion and adolescent depressive symptoms. Soc. Forces.

[18] Guan W., Kamo Y. (2015). Contextualizing Depressive Contagion. Soc. Ment. Health.

[19] Lien Y.J., Hu J.N., Chen C.Y. (2016). The influences of perceived social support and personality on trajectories of subsequent depressive symptoms in Taiwanese youth. Soc. Sci. Med..

[20] Santini Z.I., Koyanagi A., Tyrovolas S., Mason C., Haro J.M. (2015). The association between social relationships and depression: A systematic review. J. Affect. Disorders.

[21] Shelton R.C., Lee M., Brotzman L.E., Crookes D.M., Jandorf L., Erwin D., Gage-Bouchard E.A. (2019). Use of social network analysis in the development, dissemination, implementation, and sustainability of health behavior interventions for adults: A systematic review. Soc. Sci. Med..

[22] Ueno K. (2005). The effects of friendship networks on adolescent depressive symptoms. Soc. Sci. Res..

[23] Valente T.W., Pitts S.R. (2017). An appraisal of social network theory and analysis as applied to public health: Challenges and opportunities. Annu. Rev. Publ. Health.

[24] Parkhurst J.T., Hopmeyer A. (1998). Sociometric popularity and peer-perceived popularity: Two distinct dimensions of peer status. J. Early Adolesc..

[25] Okamoto J., Johnson C.A., Leventhal A., Milam J., Pentz M.A., Schwartz D., Valente T. (2011). Social Network Status and Depression among Adolescents: An Examination of Social Network Influences and Depressive Symptoms in a Chinese Sample. Res. Hum. Dev..

[26] Tucker J.S., Miles J.N.V., D’Amico E.J., Zhou A.J., Green H.D., Shih R.A. (2013). Temporal Associations of Popularity and Alcohol Use Among Middle School Students. J. Adolesc. Health.

[27] Kornienko O., Santos C.E. (2013). The Effects of Friendship Network Popularity on Depressive Symptoms During Early Adolescence: Moderation by Fear of Negative Evaluation and Gender. J. Youth Adolesc..

[28] Fujimoto K., Valente T.W. (2015). Multiplex congruity: Friendship networks and perceived popularity as correlates of adolescent alcohol use. Soc. Sci. Med..

[29] Li X., Kawachi I., Buxton O.M., Haneuse S., Onnela J.P. (2019). Social network analysis of group position, popularity, and sleep behaviors among U.S. adolescents. Soc. Sci. Med..

[30] Moody J., Brynildsen W.D., Osgood D.W., Feinberg M.E., Gest S. (2011). Popularity trajectories and substance use in early adolescence. Soc. Netw..

[31] Kamis C., Copeland M. (2020). The Long Arm of Social Integration: Gender, Adolescent Social Networks, and Adult Depressive Symptom Trajectories. J. Health Soc. Behav..

[32] Teunissen H.A., Adelman C.B., Prinstein M.J., Spijkerman R., Poelen E.A.P., Engels R.C.M.E., Scholte R.H.J. (2010). The interaction between pubertal timing and peer popularity for boys and girls: An integration of biological and interpersonal perspectives on adolescent depression. J. Abnorm. Child Psych..

[33] Lessard L.M., Juvonen J. (2018). Losing and gaining friends: Does friendship instability compromise academic functioning in middle school?. J. Sch. Psychol..

[34] Poulin F., Chan A. (2010). Friendship stability and change in childhood and adolescence. Dev. Rev..

[35] Kiuru N., Burk W.J., Laursen B., Nurmi J.-E., Salmela-Aro K. (2012). Is Depression Contagious? A Test of Alternative Peer Socialization Mechanisms of Depressive Symptoms in Adolescent Peer Networks. J. Adolesc. Health.

[36] Pachucki M.C., Ozer E.J., Barrat A., Cattuto C. (2015). Mental health and social networks in early adolescence: A dynamic study of objectively-measured social interaction behaviors. Soc. Sci. Med..

[37] Schaefer D.R., Kornienko O., Fox A.M. (2011). Misery Does Not Love Company. Am. Sociol. Rev..

[38] Van Zalk M.H.W., Kerr M., Branje S.J.T., Stattin H., Meeus W.H.J. (2010). It takes three: Selection, influence, and de-selection processes of depression in adolescent friendship networks. Dev. Psychol..

[39] Snijders T.A.B. (2016). Stochastic actor-oriented models for network change. Annu. Rev. Stat. Its Appl..

[40] Snijders T.A.B., van de Bunt G.G., Steglich C.E.G. (2010). Introduction to stochastic actor-based models for network dynamics. Soc. Netw..

[41] Christakis N.A., Fowler J.H. (2012). Social contagion theory: Examining dynamic social networks and human behavior. Stat Med..

[42] Smith K.P., Christakis N.A. (2008). Social networks and health. Annu. Rev. Sociol..

[43] Peters E., Cillessen A.H.N., Riksen-Walraven J.M., Haselager G.J.T. (2010). Best friends’ preference and popularity: Associations with aggression and prosocial behavior. Int. J. Behav. Dev..

[44] Larmer B. Inside a Chinese Test-Prep Factory. New York Times.

[45] Yang W., Xiong G., Garrido L.E., Zhang J.X., Wang M.-C., Wang C. (2018). Factor structure and criterion validity across the full scale and ten short forms of the CES-D among Chinese adolescents. Psychol. Assess..

[46] Ripley R.M., Snijders T.A.B. (2018). Manual for RSiena.

[47] R Development Core Team (2011). R: A Language and Environment for Statistical Computing.

[48] Veenstra R., Steglich C., Laursen B., Little T.D., Card N.A. (2012). Actor-based model for network and behavior dynamics: A tool to examine selection and influence processes. Handbook of Developmental Research Methods.

[49] Litwack S.D., Aikins J.W., Cillessen A.H.N. (2012). The distinct roles of sociometric and perceived popularity in friendship: Implications for adolescent depressive affect and self-esteem. J. Early Adolesc..

[50] Tan C.S., Krishnan S.A., Lee Q.W. (2017). The role of self-esteem and social support in the relationship between extraversion and happiness: A serial mediation model. Curr. Psychol..

[51] Von Dras D.D., Siegler I.C. (1997). Stability in extraversion and aspects of social support at midlife. J. Per. Soc. Psychol..

[52] Block P. (2015). Reciprocity, transitivity, and the mysterious three-cycle. Soc. Netw..

[53] McCann M., Jordan J.-A., Higgins K., Moore L. (2019). Longitudinal Social Network Analysis of Peer, Family, and School Contextual Influences on Adolescent Drinking Frequency. J. Adolesc. Health.

[54] Osgood D.W., Ragan D.T., Wallace L., Gest S.D., Feinberg M.E., Moody J. (2013). Peers and the Emergence of Alcohol Use: Influence and Selection Processes in Adolescent Friendship Networks. J. Res. Adolesc.

[55] Simone M., Long E., Lockhart G. (2017). The Dynamic Relationship between Unhealthy Weight Control and Adolescent Friendships: A Social Network Approach. J. Youth Adolesc..

[56] Dijkstra J.K., Cillessen A.H.N., Borch C. (2013). Popularity and adolescent friendship networks: Selection and influence dynamics. Dev. Psychol..

[57] Mercken L., Steglich C., Sinclair P., Holliday J., Moore L. (2012). A longitudinal social network analysis of peer influence, peer selection, and smoking behavior among adolescents in British schools. Health Psychol..

